# Trimethyl Chitosan-Engineered Cod Skin Peptide Nanosystems Alleviate Behavioral and Cognitive Deficits in D-Galactose-Induced Alzheimer’s Disease Model Mice

**DOI:** 10.3390/md23120472

**Published:** 2025-12-10

**Authors:** Songzhi Kong, Lijiao Lv, Jiaqi Guo, Guiping Lu, Dongdong Li, Xin Zhou

**Affiliations:** College of Chemistry and Environmental Science, Guangdong Ocean University, Zhanjiang 524088, China; kongsongzhi@126.com (S.K.); lvlijiao1210@163.com (L.L.); amieshuigjq@163.com (J.G.); 18216727176@163.com (G.L.); hebe502@yeah.net (D.L.)

**Keywords:** trimethyl chitosan nanoparticles, cod skin collagen peptides, Alzheimer’s disease, neuroprotection

## Abstract

Alzheimer’s disease (AD) is a common neurodegenerative disorder with limited effective treatments. Cod skin collagen peptides (CSCPs) have neuroprotective potential for AD but face poor bioavailability—due to gastrointestinal enzyme cleavage and hepatic first-pass metabolism—prompting this study to develop a nanodelivery system to enhance CSCPs’ efficacy. Trimethyl chitosan (TMC)-based CSCP-loaded nanoparticles (CSCPs-NPs) were synthesized via ionic gelation, characterized for physicochemical properties, and tested in a D-galactose-induced AD mouse model (six groups: normal control, model, CSCPs low/high dose, blank NPs, CSCPs-NPs) using behavioral tests, histopathology, immunohistochemistry, and ELISA. CSCPs-NPs had a hydrodynamic diameter of 93.25 ± 21.52 nm, polydispersity index of 0.18 ± 0.13, 61.17% encapsulation efficiency, and sustained 24 h release. In AD mice, CSCPs-NPs significantly improved cognitive function and motor coordination, reduced hippocampal atrophy, preserved neurons, and mitigated oxidative stress, neuroinflammation, and apoptosis (upregulated Bcl-2, downregulated Bax)—effects matching high-dose free CSCPs. This TMC-based nanoformulation enhances CSCPs’ bioavailability and provides a promising strategy for AD intervention.

## 1. Introduction

Alzheimer’s disease (AD) is a major challenge in modern neurology and aging research. As the most common form of dementia, it affects over 50 million people globally, with projections of 150 million cases by 2050 [1,2]. This progressive neurodegenerative disorder causes cognitive decline, memory loss, and eventual functional impairment, placing heavy burdens on healthcare systems, caregivers, and societies worldwide [3]. Pathological hallmarks include extracellular amyloid-beta (Aβ) plaques, intracellular neurofibrillary tangles of hyperphosphorylated tau, synaptic dysfunction, and neuronal death [4,5,6]. Despite decades of research, current therapies remain symptomatic, offering modest temporary relief without slowing disease progression. Repeated failures of anti-Aβ therapies in clinical trials highlight AD pathogenesis complexity and the need for novel strategies [6,7].

AD’s multifactorial nature suggests effective interventions require multi-target approaches. Oxidative stress is a critical factor in AD pathogenesis [8]. The brain’s high oxygen consumption, abundant polyunsaturated fatty acids, and weak antioxidant defenses make it vulnerable to oxidative damage. Studies show elevated oxidative stress markers and impaired antioxidant systems in AD patients and animal models [8,9,10]. This oxidative environment damages neurons via lipid peroxidation, protein oxidation, and DNA damage, leading to synaptic dysfunction and cell death. Neuroinflammation also drives AD progression [11]. Chronic microglia and astrocyte activation releases pro-inflammatory cytokines, creating a toxic environment that worsens neuronal damage and impairs repair [12].

Natural bioactive compounds have gained attention as potential neuroprotective agents due to their multi-target effects, favorable safety, and long history of human use [13,14,15]. Marine-derived peptides, especially from fish collagen, show antioxidant, anti-inflammatory, and neuroprotective activities [16,17,18]. Cod (*Gadus morhua*) skin, a major byproduct from commercial fishing and processing industries, represents an abundant, sustainable, and cost-effective raw material for collagen extraction. This marine collagen exhibits unique physicochemical properties including high solubility, excellent thermal stability, and a distinctive amino acid profile rich in glycine, proline, and hydroxyproline [19,20,21]. Enzymatic hydrolysis of cod skin collagen yields peptides with enhanced bioavailability and bioactivity [22,23,24], characterized by strong specificity, low toxicity, and high antioxidant capacity–properties that are particularly valuable for the development of therapeutics targeting oxidative stress-related diseases [23,24,25]. These peptides can effectively scavenge free radicals, reduce inflammation, and protect neurons from various insults [23,24,25]. However, their use in neurological disorders is limited by poor in vivo performance: oral administration exposes them to cleavage by gastrointestinal enzymes (e.g., pepsin, trypsin), hindering transmembrane transport across tight epithelial layers. They also undergo extensive hepatic first-pass metabolism, drastically reducing intact peptide levels in systemic circulation. This compromises peptide integrity at target sites, leading to low bioavailability.

Nanotechnology-based delivery systems offer solutions to these issues. Among nanocarriers, chitosan-based nanoparticles are attractive for their biocompatibility, biodegradability, low toxicity, and mucoadhesive properties [26]. Trimethyl chitosan (TMC)—a quaternized chitosan derivative—has better solubility across pH ranges and enhanced permeation compared to unmodified chitosan [27]. These traits make TMC nanoparticles suitable for delivering therapeutics across biological barriers. Their positive surface charge facilitates interaction with negatively charged cell membranes, potentially boosting cellular uptake and bioavailability of encapsulated compounds.

While collagen peptides and chitosan nanoparticles have been studied individually, combining cod skin collagen peptides with TMC nanoparticles for AD treatment remains unexplored. This study develops a novel nanoformulation of cod skin collagen peptides (CSCPs) encapsulated in TMC nanoparticles (CSCPs-NPs) and evaluates its therapeutic potential in a D-galactose-induced aging mouse model—one that shares pathological features with AD. D-galactose administration is widely used to induce accelerated aging, characterized by oxidative stress, neuroinflammation, and cognitive impairment, providing a relevant platform to test potential AD therapeutics.

We hypothesize CSCPs-NPs will show enhanced neuroprotective effects compared to free CSCPs, due to improved stability, bioavailability, and possibly better brain delivery. Through behavioral assessments, histopathological examinations, and molecular analyses, we aim to clarify the mechanisms behind CSCPs-NPs’ therapeutic effects, focusing on oxidative stress pathways, inflammatory responses, and apoptotic processes. Findings from this study may offer insights into developing novel nanoformulations for AD treatment and advance the field of nutraceutical-based therapies for neurodegenerative diseases.

## 2. Results

### 2.1. Preparation and Characterization of CSCPs-NPs

**Figure 1:**
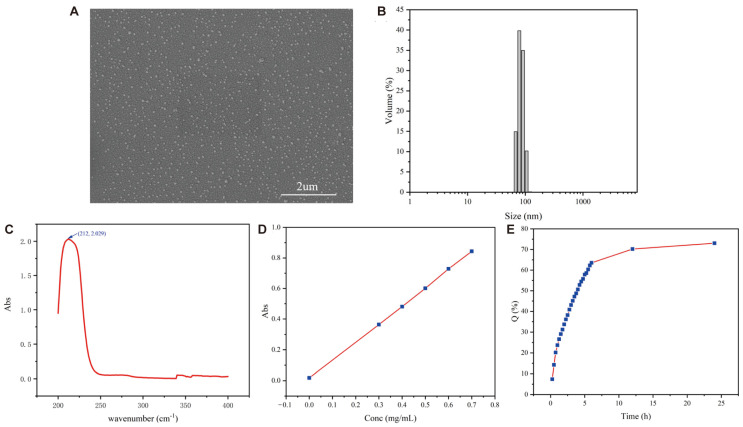
Preparation and characterization of CSCPs-NPs. (**A**) Scanning electron microscope (SEM) micrograph showing spherical morphology of lyophilized nanoparticles. (**B**) Hydrodynamic size distribution of CSCPs-NPs measured by dynamic light scattering (DLS). (**C**,**D**) UV absorption curve (**C**) and standard curve (**D**) of CSCPs. (**E**) In vitro release profile of CSCPs from NPs over 24 h in PBS (pH 7.4) at 37 °C.

CSCPs-NPs were fabricated using a two-step strategy involving quaternization of chitosan followed by ionic gelation-mediated peptide encapsulation. Briefly, medium-molecular-weight chitosan (200 kDa, 90% deacetylation) was reacted with dimethyl sulfate under controlled alkaline conditions at 40 °C for 5 h under nitrogen atmosphere, yielding trimethyl chitosan (TMC) with a quaternization degree of 57.3% as determined by ^1^H-NMR (Figure S1). The resulting TMC was then complexed with CSCPs (4.2 mg/mL) at a mass ratio of 1:2.1 under gentle stirring (500 rpm, 10 min), facilitating electrostatic self-assembly into nanometric particles (Figure 1A and Figure S2). Post-lyophilization with sucrose as cryoprotectant, the nanoparticles exhibited excellent redispersibility and storage stability over 90 days at 4 °C.

Dynamic light scattering (DLS) analysis confirmed a narrow size distribution with an average hydrodynamic diameter of 93.25 ± 21.52 nm and a polydispersity index (PDI) of 0.18 ± 0.13 (Figure 1B). Using UV-Vis spectrophotometry calibrated with a standard curve (R^2^ = 0.9997) (Figure 1C,D), the encapsulation efficiency and drug loading capacity were determined to be 61.17% and 17.53%, respectively. In vitro release studies demonstrated sustained CSCP release over 24 h without an initial burst effect: 28.68% at 1 h, 63.52% at 6 h, and 73.05% at 24 h (Figure 1E).

### 2.2. CSCPs-NPs Mitigate Hippocampal Atrophy and Neuronal Damage

A D-galactose-induced AD model was established in KM mice via chronic subcutaneous administration. Although no significant differences in whole-brain weight or volume were observed among groups, high-resolution histopathological scanning revealed notable unilateral or bilateral hippocampal atrophy in model mice, which was markedly alleviated by CSCPs-NPs treatment (Figure S3).

**Figure 2:**
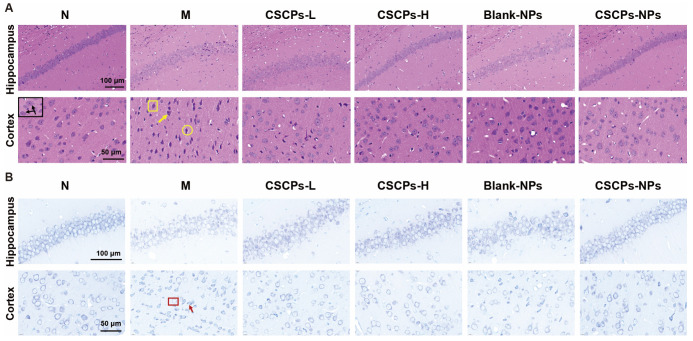
CSCPs-NPs attenuate hippocampal atrophy and neuronal damage in D-galactose-induced AD mice. (**A**) Representative HE staining of the hippocampus and cerebral cortex in brain tissue of mice. (Black arrows: normal neuron morphology; yellow circle: karyopyknosis; yellow arrow: edema; yellow box: nuclear deviation or disappearance). (**B**) Representative Nissl staining images of the hippocampus and cerebral cortex in brain tissue of mice. (Red box: neuronal loss; red arrow: karyopyknosis).

H&E and Nissl staining further demonstrated severe neuronal disorganization, karyopyknosis, edema, and neuronal loss in the hippocampus and cortex of model mice (Figure 2A,B). In contrast, CSCPs-NPs administration preserved cytoarchitecture, increased neuronal density, and reduced vacuolation and nuclear shrinkage. Notably, CSCPs-L treatment failed to rescue neuronal damage, whereas CSCPs-H and CSCPs-NPs showed comparable neuroprotective effects.

### 2.3. CSCPs-NPs Improve Cognitive and Motor Functions in AD Mice

**Figure 3:**
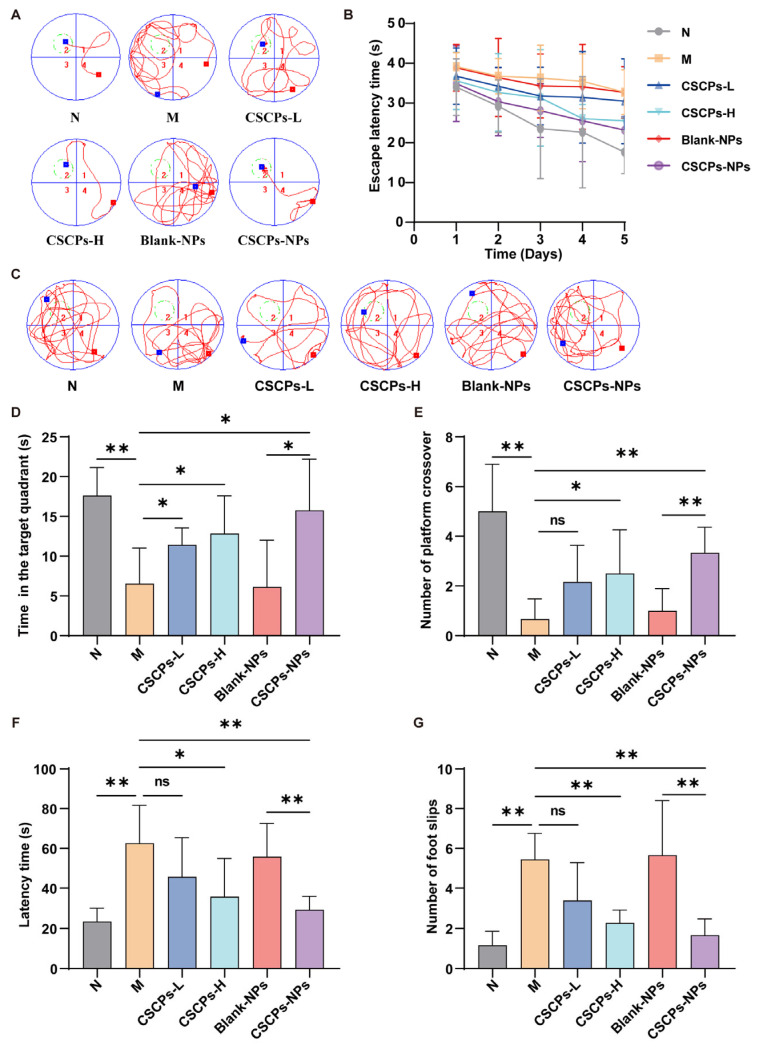
CSCPs-NPs improve cognitive and motor functions in AD mice. (**A**) Procedure diagram for spatial learning and travel path tracings of mice in Morris water maze training. The red square denotes the starting point of the mouse’s path, the blue square the end point, the red line its swimming trajectory, and the green circle the platform’s location. (**B**) Escape latency during the 5-day training period. (**C**) Procedure diagram for spatial memory test and travel path tracings of mice in probe trial of Morris water maze. (**D**) Time spent in the target quadrant during the probe trial. (**E**) Number of platform crossings. (**F**) Time taken to traverse the balance beam. (**G**) Number of foot slips during the beam traversal. Data are presented as mean ± SD (*n* = 6). * *p* < 0.05, ** *p* < 0.01. “ns” indicates no significant difference.

Behavioral tests were conducted to evaluate spatial learning, memory, and motor coordination. In the Morris water maze, CSCPs-NPs-treated mice exhibited significantly shortened escape latency during training (Figure 3A,B) and spent more time in the target quadrant during the probe test (Figure 3C,D), indicating enhanced spatial memory. They also showed increased platform crossings (Figure 3E). In the balance beam test, the CSCPs-NPs group displayed reduced traversal time and fewer foot slips (Figure 3F,G), demonstrating improved motor coordination and balance compared to model mice. CSCPs-L did not improve behavioral deficits, while CSCPs-H and CSCPs-NPs produced similar improvements in cognitive and motor functions. These results indicated that CSCPs-NPs treatment in AD mice leads to improvements in cognitive function and motor performance.

### 2.4. CSCPs-NPs Restore Apoptotic Balance in the Hippocampus

**Figure 4:**
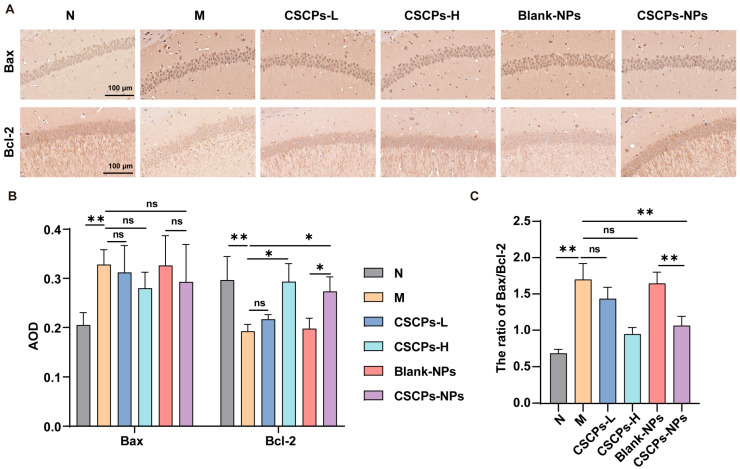
CSCPs-NPs restore apoptotic balance in the hippocampus of AD mice. (**A**) Representative immunohistochemical images showing Bcl-2 and Bax expression in hippocampus. (**B**,**C**) Quantitative analysis of Bcl-2 and Bax expression levels (**B**) and the calculated Bax/Bcl-2 ratio (**C**) by IHC. * *p* < 0.05, ** *p* < 0.01. “ns” indicates no significant difference.

To decipher the underpinning mechanism at the molecular level, we further assessed the expression of the key apoptotic regulators Bcl-2 and Bax. Immunohistochemical analyses revealed that D-galactose induction significantly dysregulated apoptosis-related proteins, suppressing Bcl-2 and elevating Bax expression, resulting in a high Bax/Bcl-2 ratio (Figure 4A–C). Treatment with CSCPs-NPs substantially reversed these changes, upregulating Bcl-2 and downregulating Bax, thereby normalizing the Bax/Bcl-2 ratio. CSCPs-H showed similar efficacy, while CSCPs-L and Blank-NPs had no significant effects. These results indicate that CSCPs-NPs mitigate neuronal apoptosis and contribute to neuroprotection in AD mice.

### 2.5. CSCPs-NPs Alleviate Oxidative Stress and Neuroinflammation via ROS/NF-κB Pathway

**Figure 5:**
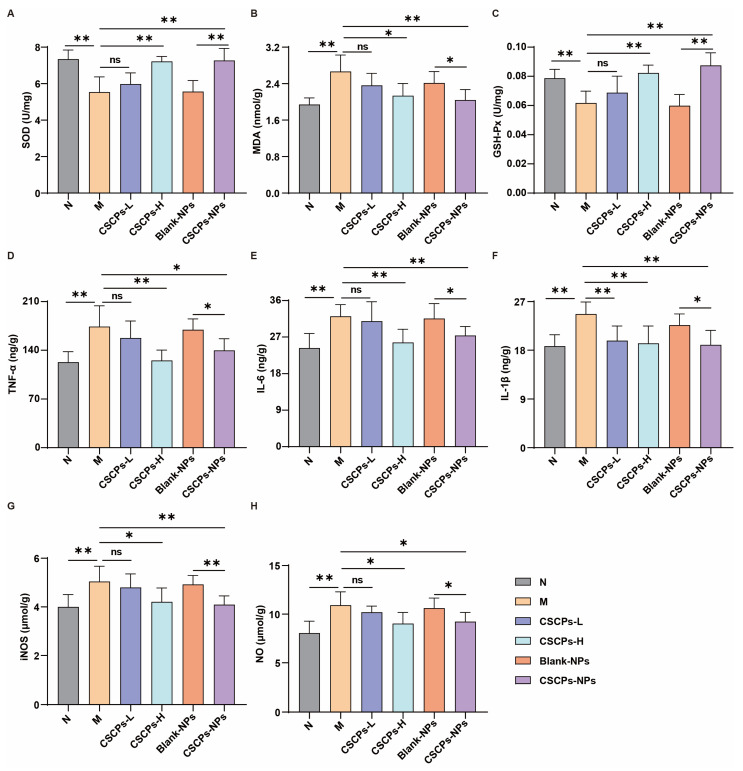
CSCPs-NPs attenuate oxidative stress and neuroinflammation through the ROS/NF-κB pathway. (**A**–**C**) Oxidative stress parameters: (**A**) SOD activity, (**B**) GSH-Px level, (**C**) MDA content. (**D**–**F**) Pro-inflammatory cytokines: (**D**) TNF-α, (**E**) IL-6, (**F**) IL-1β. (**G**,**H**) NF-κB pathway markers: (**G**) iNOS expression and (**H**) NO content. Values are mean ± SD (n = 6). * *p* < 0.05, ** *p* < 0.01. “ns” indicates no significant difference.

Given the interplay between oxidative stress and neuroinflammation in AD progression, we evaluated whether CSCPs-NPs alleviate D-galactose-induced damage through redox and inflammatory modulation. CSCPs-NPs significantly attenuated oxidative stress in brain tissue, as evidenced by increased SOD activity and GSH-Px levels, alongside reduced MDA content (Figure 5A–C). Concurrently, CSCPs-NPs suppressed neuroinflammation, markedly reducing pro-inflammatory cytokine levels (TNF-α, IL-6, and IL-1β; Figure 5D–F). Mechanistic studies revealed inhibition of ROS-induced NF-κB activation, shown by reduced nuclear translocation of p65 and decreased expression of iNOS and NO (Figure 5G,H). CSCPs-H produced comparable effects, whereas CSCPs-L and Blank-NPs showed minimal impacts. These findings underscore the role of CSCPs-NPs in modulating the ROS/NF-κB pathway to counteract oxidative stress and inflammation in AD.

## 3. Discussion

Developing effective therapeutic strategies for Alzheimer’s disease (AD) remains a critical challenge in neurological research. In this study, we designed, synthesized, and evaluated a novel nanodelivery system—trimethyl chitosan (TMC)-based nanoparticles loaded with cod skin collagen peptides (CSCPs-NPs)—which significantly alleviated cognitive decline, neuronal damage, and neuroinflammation in a D-galactose-induced mouse model of AD.

A central finding of this work is the successful fabrication of CSCPs-NPs with favorable physicochemical properties: uniform nanosize (~93 nm), positive surface charge, and sustained release kinetics without an initial burst effect. These traits support prolonged neuroprotective action, as sustained release maintains steady peptide levels at target sites and avoids concentration fluctuations that limit therapeutic efficacy. Incorporation of TMC—a quaternized chitosan derivative—provides a new approach to advancing peptide-based strategies for the prevention and treatment of neurological disorders. First, TMC enhanced mucoadhesion and cellular uptake, facilitating peptide transit across epithelial layers that typically impede transmembrane transport of free peptides. Second, TMC encapsulation is expected to improve CSCP stability in physiological environments, as it is known to shield peptides from cleavage by gastrointestinal enzymes (e.g., pepsin, trypsin) and reduce exposure to hepatic first-pass metabolism—two major causes of low bioavailability for oral peptide formulations [28,29]. Our formulation achieved 61.17% encapsulation efficiency and sustained CSCP release over 24 h, supporting its potential to reduce dosing frequency and improve patient compliance by maintaining therapeutic peptide levels without high, frequent doses of free CSCPs.

Notably, CSCPs-NPs exhibited significant neurorestorative effects. Histopathological analyses showed marked reductions in hippocampal atrophy and neuronal loss, supported by improved performance in the Morris water maze and balance beam tests—confirming the formulation’s ability to mitigate AD-related pathological and functional deficits. A key observation is the efficacy difference between free and encapsulated CSCPs: the low-dose free CSCP group (CSCPs-L) had negligible therapeutic effects, while CSCPs-NPs—administered at an equivalent peptide dose—produced outcomes similar to the high-dose free CSCP group (CSCPs-H). This indicates nanoencapsulation drastically enhanced CSCPs’ bioavailability without altering the peptide’s intrinsic neuroprotective properties.

At the molecular level, CSCPs-NPs acted via multi-target mechanisms addressing key AD pathological pathways. Upregulation of Bcl-2 and downregulation of Bax (reducing the Bax/Bcl-2 ratio) indicate CSCPs-NPs mitigate mitochondrial apoptosis—a well-established driver of neuronal loss in AD [30,31]. Additionally, the nanoparticles significantly attenuated oxidative stress, as shown by increased SOD and GSH-Px activity and decreased MDA levels. They also suppressed neuroinflammation via inhibition of the ROS/NF-κB pathway, reducing production of pro-inflammatory cytokines (TNF-α, IL-6, IL-1β) and downregulating iNOS and NO synthesis. This triple mechanism—anti-apoptotic, antioxidant, anti-inflammatory—highlights the pleiotropic neuroprotective effects of CSCPs when delivered via a tailored nanocarrier.

Compared to existing peptide delivery systems, TMC offers distinct advantages: biodegradability, low toxicity, and ease of modification. Unlike synthetic polymers, TMC is derived from natural chitosan, making it more suitable for long-term therapy [24]. Moreover, the ionic gelation method used here is scalable, reproducible, and avoids organic solvents—enhancing its translational potential.

Nevertheless, this study has several limitations. While the D-galactose model is well-accepted for studying aging and AD-like pathology, it does not fully recapitulate the amyloid-beta or tau pathology characteristic of human AD. Future studies should validate these findings in transgenic AD models. The formulation optimization process, though informed by preliminary screening and established nanoparticle literature, was not exhaustively explored using high-throughput or statistical design approaches; future work would benefit from systematic optimization of critical parameters such as TMC and CSCP concentrations and mass ratios to further improve encapsulation efficiency and stability. Moreover, direct experimental validation of formulation stability under physiological conditions—particularly in simulated gastric and intestinal fluids—was not performed here, though such testing will be essential in future studies to fully confirm the protective effects of TMC encapsulation. Additionally, accelerated stability studies under controlled stress conditions (e.g., elevated temperature and humidity) were not conducted; these will be important to establish the formulation’s shelf-life and storage stability. Finally, while we demonstrated CSCPs-NPs modulate the NF-κB pathway, the precise intracellular signaling cascades and receptor-level interactions remain to be elucidated.

In conclusion, we developed a biocompatible, efficacious CSCP nanoformulation that overcomes the limitations of peptide therapeutics. CSCPs-NPs significantly improved behavioral and biochemical outcomes in AD mice via multi-target modulation of apoptosis, oxidative stress, and neuroinflammation. These results underscore the potential of nanotechnology-enabled peptide delivery as a strategy for neurodegenerative diseases. Future work will focus on detailed pharmacokinetics, long-term toxicity, and efficacy in additional animal models to support clinical translation.

## 4. Materials and Methods

### 4.1. Synthesis of Trimethyl Chitosan (TMC)

TMC was synthesized from medium-molecular-weight chitosan (200 kDa, 90% deacetylation; Macklin, Shanghai, China; CAS: 9012-76-4) using dimethyl sulfate (98%, Macklin, CAS: 77-78-1) as the quaternizing agent, following a previously described method with modifications [32]. Briefly, 0.85 g of chitosan was dispersed in 15 mL deionized water containing 0.88 g NaCl under constant stirring at 40 °C for 10 min. Then, 16 mL of dimethyl sulfate was added, followed by dropwise addition of 10 mL NaOH solution (1.2 g in deionized water). The reaction proceeded for 5 h under magnetic stirring, yielding a yellow viscous mixture. The product was concentrated by rotary evaporation, dialyzed (MWCO: 8–1.4 kDa, Shanghai yuanye, Shanghai, China) against deionized water for 3 days with water changes every 12 h, and further concentrated to one-third of the original volume. The concentrate was precipitated in absolute ethanol for 24 h, filtered, and washed twice with ether and ethanol. The final product was lyophilized and stored at −20 °C.

### 4.2. Preparation and Characterization of CSCP-Loaded TMC Nanoparticles (CSCPs-NPs)

The preparation of CSCP-loaded TMC nanoparticles was adapted from the ionic cross-linking method described by Yuan et al. [33]. Briefly, CSCPs-NPs were prepared using an ionic cross-linking method. Solution A was prepared by dissolving 10 mg TMC and 21 mg food-grade CSCPs (Zhongshiduqing (Shandong) Biotechnology Co., LTD, Heze, China) in 5 mL ultrapure water under stirring at 25 °C. Solution B contained 1.2 mg sodium tripolyphosphate (TPP, AR 98%, Macklin, CAS: 7758-29-4, AR 98%, Macklin, CAS: 7758-29-4) and 0.6 mg sodium alginate (AR 90%, Macklin, CAS: 9005-38-3) dissolved in 2 mL ultrapure water. Solution B was added dropwise (1 mL/min) to solution A under magnetic stirring at 500 rpm for 10 min. The resulting nanoparticles were lyophilized with sucrose (5% *w*/*v*) as a cryoprotectant. Particle size, polydispersity index (PDI), and zeta potential were measured using a Zetasizer Nano ZS (Malvern Panalytical, Malvern, Worcestershire, UK). Encapsulation efficiency (EE) and drug loading (DL) were determined by UV-Vis spectrophotometry at 212 nm.

### 4.3. In Vitro Release Study

The in vitro release profile of CSCPs from nanoparticles was evaluated using a dialysis method. Lyophilized CSCPs-NPs were reconstituted, centrifuged, and the pellet transferred into a dialysis bag (MWCO: 8–1.4 kDa). The bag was immersed in 30 mL PBS (pH 7.4) and stirred at 100 rpm at 37 °C. At predetermined time points, 2 mL of release medium was sampled and replaced with an equal volume of fresh PBS. The cumulative release percentage was calculated using the formula:

$$Q\;(\%)\;=\;\frac{V_{e}\sum_{1}^{n-1}C_{i}+V_{0}C_{n}}{m_{\mathrm{drug}}}\times 100\%\;$$

where (*V_e_*) is the sample volume, (*V*_0_) is the total release medium volume, (*C_i_*) is the concentration at the i-th sampling, (*m_drug_*) is the total drug content, and (*_n_*) is the number of samplings.

### 4.4. Animals and Drug Administration

Male Kunming mice (6–8 weeks old, 35–45 g) were obtained from the Guangdong Medical Laboratory Animal Center (China) and housed under standard conditions (23 ± 2 °C, 12 h light/dark cycle) with free access to food and water. Animal experimental procedures were in line with National Institutes of Health Guide for Care and Use of Laboratory Animals and approved by the ethics committee of the animal laboratory of Guangdong Ocean University. After one week of acclimatization, mice were randomly divided into six groups (n = 9): normal control (administered saline), model (given D-galactose at 200 mg/kg/day via subcutaneous injection [s.c.]), CSCPs-L (D-galactose 200 mg/kg/day s.c. plus CSCPs at 1 g/kg/day via oral gavage [p.o.]), CSCPs-H (D-galactose 200 mg/kg/day s.c. plus CSCPs at 2 g/kg/day p.o.), blank-NPs (D-galactose 200 mg/kg/day s.c. plus blank nanoparticles at 10 mL/kg/day p.o.), and CSCPs-NPs (D-galactose 200 mg/kg/day s.c. plus CSCPs-NPs containing 1 g CSCPs/kg/day p.o.), with all treatments administered for 10 weeks.

### 4.5. Behavioral Tests

Morris Water Maze (MWM): Spatial learning and memory were evaluated using a circular water tank (110 cm in diameter) containing opaque water maintained at 22 °C. Distinct visual cues were positioned around the pool. During the five-day acquisition phase, animals were trained to find a submerged platform (8 cm diameter, 1 cm below water surface). Each animal completed four trials daily with a 1 h intertrial rest period. Escape latency and swim velocity were recorded. A probe trial was performed on day 6 in which the platform was removed; time spent in the target quadrant and number of platform crossings were analyzed with MWM software (Labmaze V1.0, Beijing Zhongshi Dachuang Technology Development Co., Ltd., Beijing, China).

Balance Beam Test: Sensorimotor coordination was assessed using a narrow beam (1 cm wide) positioned 44 cm above a padded surface. Mice were placed at one end and encouraged to cross to a darkened goal box. Each mouse was given three trials per day. Latency to cross and number of foot slips were recorded. Animals that failed to cross within 60 s were assigned the maximum latency.

### 4.6. Histopathological Examination

Brain samples were fixed in 4% paraformaldehyde, embedded in paraffin, and cut into 4 μm sections. Tissue sections were stained with hematoxylin and eosin (H&E) and Nissl stain to assess neuronal cytoarchitecture and the status of Nissl bodies.

### 4.7. Immunohistochemistry (IHC)

Deparaffinized sections were incubated with primary antibodies against Bcl-2 (1:200, Biosynthesis) and Bax (1:200, Boster) overnight at 4 °C. After washing, HRP-conjugated secondary antibodies were applied. Color development was carried out using DAB, and sections were counterstained with hematoxylin. Stained sections were imaged and quantitatively analyzed using ImageJ 1.53e. Integrated optical density (IOD) and average optical density (AOD) were determined from three randomly selected sections per animal.

### 4.8. ELISA

Following the 10-week treatment period, brain tissues were homogenized in cold saline (1:9, *w*/*v*) and centrifuged at 2500 rpm for 10 min at 4 °C. The supernatants were assayed for oxidative stress markers (SOD, MDA, GSH-Px), inflammatory cytokines (TNF-α, IL-1β, IL-6), and markers related to NF-κB signaling (NO, iNOS) using commercial ELISA kits (Nanjing Jiancheng Bioengineering Institute, Nanjing, China) according to the manufacturer’s protocols.

### 4.9. Statistical Analysis

Data are presented as mean ± standard deviation (SD). Differences between two groups were assessed using Student’s *t*-test. Multiple group comparisons were conducted using one-way or two-way ANOVA followed by Tukey’s post hoc test. All statistical operations were performed using GraphPad Prism version 9.3, and a *p*-value less than 0.05 was considered statistically significant.

## Data Availability

The data that support the findings of this study are available from the corresponding author upon reasonable request.

## Supplementary Materials

The following supporting information can be downloaded at: https://www.mdpi.com/article/10.3390/md23120472/s1, Figure S1. Structural Characterization of Trimethyl Chitosan (TMC); Figure S2. Characterization of CSCPs-NPs; Figure S3. Representative Nissl staining images of the brains from each group of mice.

## Abbreviations

The following abbreviations are used in this manuscript:

AD	Alzheimer’s disease
CSCPs	Cod skin collagen peptides
TMC	Trimethyl chitosan
CSCPs-NPs	Trimethyl chitosan (TMC)-based CSCP-loaded nanoparticles
