# Supplementary material for: Trimethyl Chitosan-Engineered Cod Skin Peptide Nanosystems Alleviate Behavioral and Cognitive Deficits in D-Galactose-Induced Alzheimer’s Disease Model Mice
Source: Mar Drugs. 2025 Dec 10;23(12):472. doi: 10.3390/md23120472 (PMC12734884; doi:10.3390/md23120472)
Supplement: Supplementary file 1 [file marinedrugs-23-00472-s001.zip › marinedrugs-3971142-supplementary.pdf]

Supplementary Figures

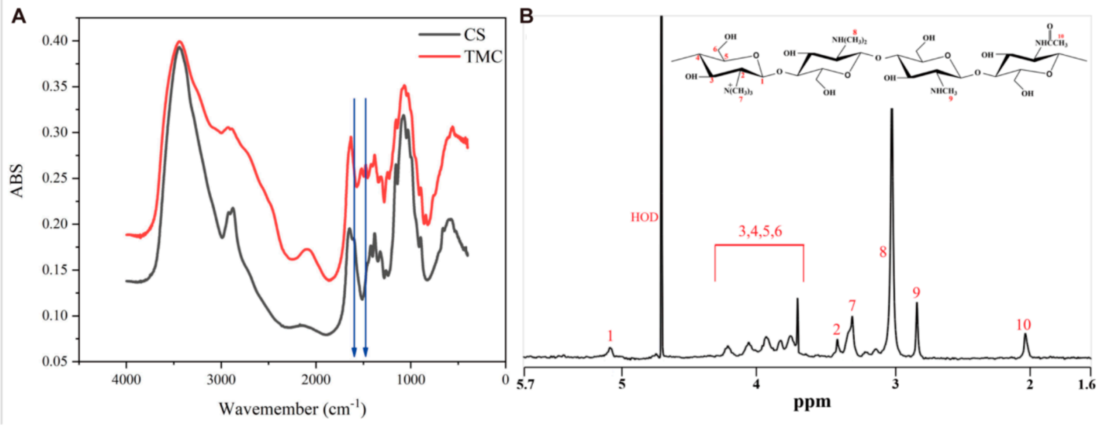

Figure S1. Structural Characterization of Trimethyl Chitosan (TMC). (A) FT-IR spectra of chitosan (CS) and synthesized TMC. The attenuation of N-H bending vibration at 1601 cm^{-1} and emergence of -N^{+}(CH$_3$)$_3$ asymmetric stretching at 1475 cm^{-1} confirm quaternization. (B) ^{1}H-NMR spectrum of TMC (D$_2$O, 300 MHz). Degree of quaternization (57.3%) calculated from integral ratios of -N^{+}(CH$_3$)$_3$ protons (δ 3.3 ppm) versus H-1 of glucosamine (δ 4.7~5.7 ppm).

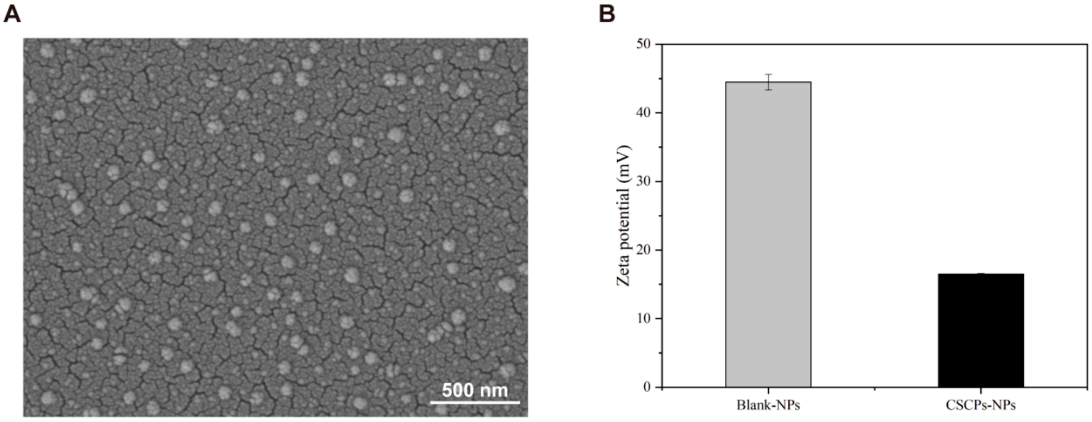

Figure S2. Characterization of CSCPs-NPs. (A) Scanning electron microscope (SEM) micrograph showing spherical morphology of lyophilized nanoparticles. (B) Zeta potential of CSCPs-NPs.

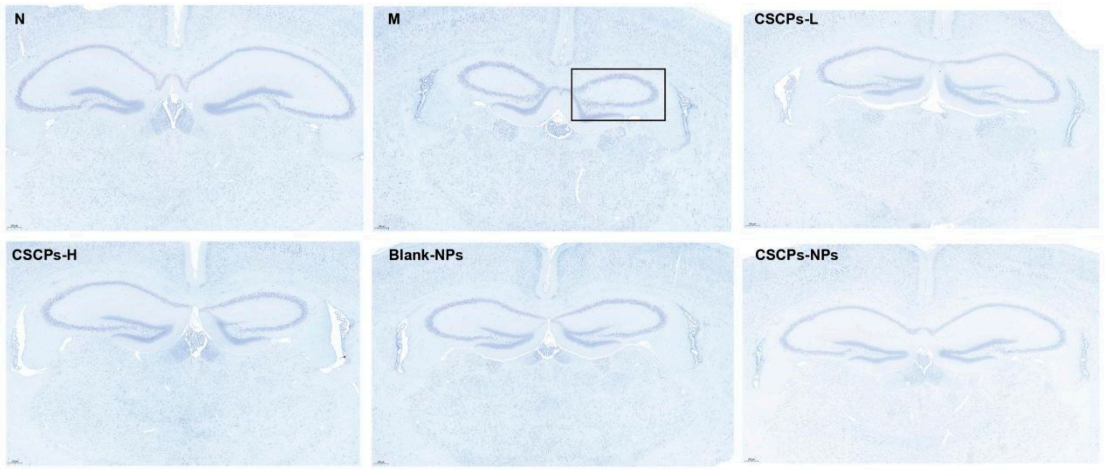

Figure S3. Representative Nissl staining images of the brains from each group of mice. (Black boxes indicate regions of hippocampal atrophy)
